# Glutamate connectivity associations converge upon the salience network in schizophrenia and healthy controls

**DOI:** 10.1038/s41398-021-01455-y

**Published:** 2021-05-27

**Authors:** Robert A. McCutcheon, Toby Pillinger, Maria Rogdaki, Juan Bustillo, Oliver D. Howes

**Affiliations:** 1 Department of Psychosis Studies, Institute of Psychiatry, Psychology & Neuroscience, Kings College London, London, SE5 8AF UK; 2 Psychiatric Imaging Group, MRC London Institute of Medical Sciences, Hammersmith Hospital, London, W12 0NN UK; 3 Institute of Clinical Sciences, Faculty of Medicine, Imperial College London, London, W12 0NN UK; 4 South London and Maudsley NHS Foundation Trust, London, UK; 5 Department of Psychiatry, University of New Mexico, Albuquerque, NM USA; 6 Department of Neurosciences, University of New Mexico, Albuquerque, NM USA

**Keywords:** Pathogenesis, Molecular neuroscience

## Abstract

Alterations in cortical inter-areal functional connectivity, and aberrant glutamatergic signalling are implicated in the pathophysiology of schizophrenia but the relationship between the two is unclear. We used multimodal imaging to identify areas of convergence between the two systems. Two separate cohorts were examined, comprising 195 participants in total. All participants received resting state functional MRI to characterise functional brain networks and proton magnetic resonance spectroscopy (1H-MRS) to measure glutamate concentrations in the frontal cortex. Study A investigated the relationship between frontal cortex glutamate concentrations and network connectivity in individuals with schizophrenia and healthy controls. Study B also used 1H-MRS, and scanned individuals with schizophrenia and healthy controls before and after a challenge with the glutamatergic modulator riluzole, to investigate the relationship between changes in glutamate concentrations and changes in network connectivity. In both studies the network based statistic was used to probe associations between glutamate and connectivity, and glutamate associated networks were then characterised in terms of their overlap with canonical functional networks. Study A involved 76 individuals with schizophrenia and 82 controls, and identified a functional network negatively associated with glutamate concentrations that was concentrated within the salience network (*p* < 0.05) and did not differ significantly between patients and controls (*p* > 0.85). Study B involved 19 individuals with schizophrenia and 17 controls and found that increases in glutamate concentrations induced by riluzole were linked to increases in connectivity localised to the salience network (*p* < 0.05), and the relationship did not differ between patients and controls (*p* > 0.4). Frontal cortex glutamate concentrations are associated with inter-areal functional connectivity of a network that localises to the salience network. Changes in network connectivity in response to glutamate modulation show an opposite effect compared to the relationship observed at baseline, which may complicate pharmacological attempts to simultaneously correct glutamatergic and connectivity aberrations.

## Introduction

Dysfunction of glutamatergic signalling has been proposed as central to the pathophysiology of schizophrenia^1,2^. This link has been made on the basis of animal work, post-mortem findings, and the fact that drugs which affect glutamate signalling have the ability to both provoke and attenuate psychotic symptoms^1–3^. More recently, in vivo neuroimaging studies undertaken in individuals with schizophrenia have directly demonstrated abnormalities of cortical glutamatergic function^4^. A parallel line of research has simultaneously demonstrated that functional brain networks, as inferred from the correlation of time courses derived from resting state functional MRI (rs-fMRI), show aberrant organisation in schizophrenia^5^. Disruption of the salience network, centred upon the bilateral insula and anterior cingulate cortex, has in particular been suggested as a key mechanism underlying psychotic symptoms^6–9^. It is unclear, however, whether glutamate signalling is related to the architecture of functional brain networks.

Proton magnetic resonance spectroscopy (^1^H-MRS) allows for the in vivo quantification of brain glutamate concentrations. Findings in individuals with schizophrenia are heterogenous and are affected by region studied, illness stage, and medication^4^. Glutamatergic signalling contributes to the balance between excitation and inhibition, which generates synchronised neural oscillations^10,11^. These oscillations in turn underlie slow fluctuations of neural activity, which are observable using rs-fMRI, and the co-activation patterns generate well characterised functional brain networks^12,13^. Given the role that glutamate signalling plays in orchestrating synchronised neural activity across the brain, disruptions to glutamatergic signalling may affect functional brain networks. In keeping with this, altered connectivity of functional brain networks has been observed following the administration of drugs such as ketamine that affect glutamatergic signalling^14–16^.

Studies of functional connectivity have repeatedly highlighted aberrant network architecture in schizophrenia, and altered connectivity of the salience network appears to be of particular pathophysiological importance^6–9^. The factors underlying aberrant functional connectivity in schizophrenia are not fully understood, although alterations in synaptic signalling may play a fundamental role here. Understanding the relationship between glutamate signalling and functional brain networks is important both for an improved understanding of pathophysiological mechanisms and for a rational approach to developing pharmacological interventions^17^.

In the current study, we first used ^1^H-MRS to measure anterior cingulate cortex glutamate concentrations and rs-fMRI to measure connectivity of brain networks in individuals with schizophrenia and healthy controls. We hypothesised that glutamate concentrations would be associated with network connectivity, and given the exploratory nature of the study we used the network based statistic (NBS) to investigate connectivity across the entire cortex. We next tested the hypothesis that glutamate associated networks would preferentially overlap the salience network. We finally investigated whether pharmacologically induced perturbations of glutamatergic signalling in individuals with schizophrenia and healthy controls was linked to perturbations of cortical connectivity, and whether this too localised to the salience network.

## Methods and materials

**Fig. 1:**
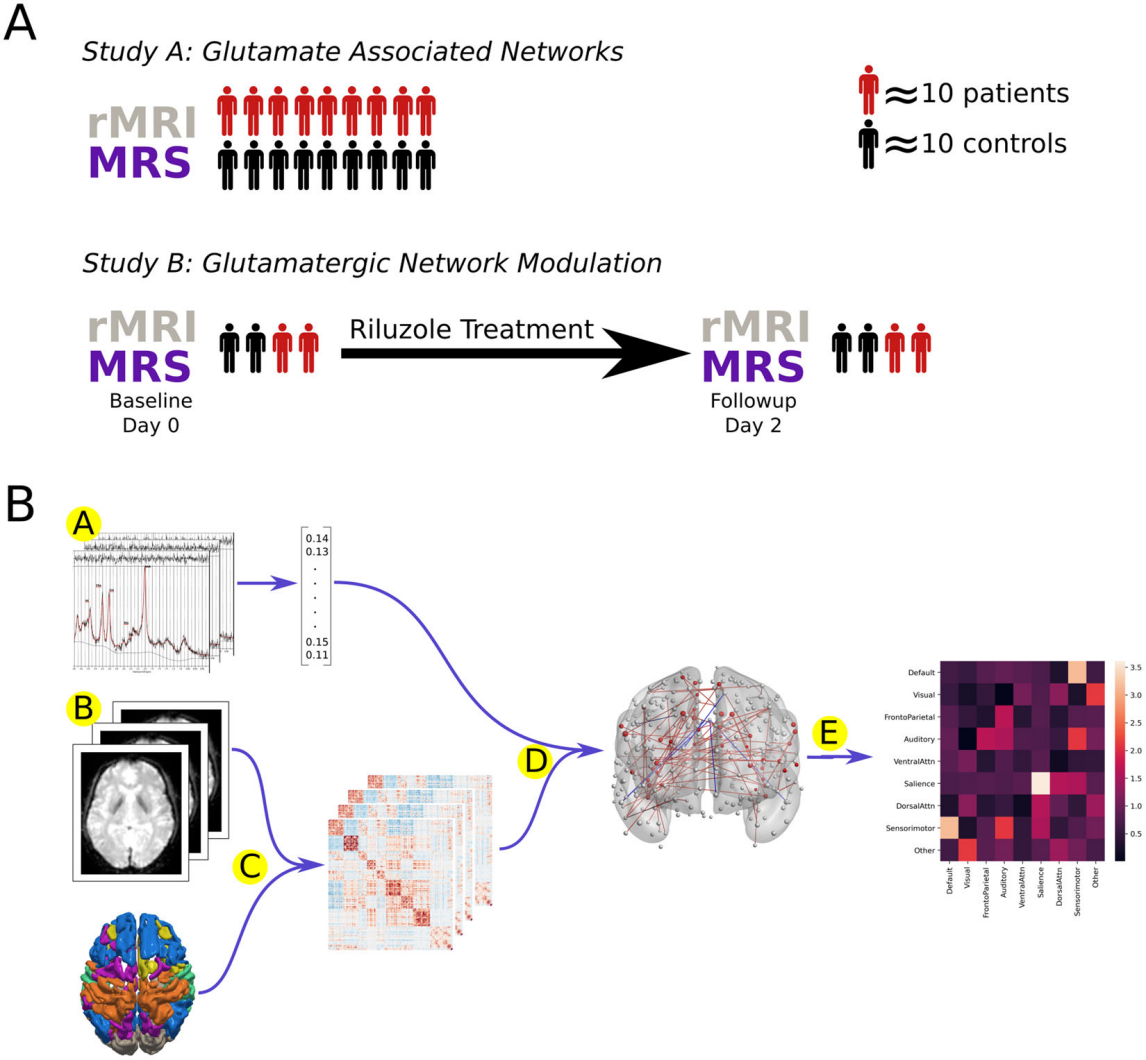
Overview of methods. **A** Two cohorts of subjects are reported, Study A consists of patients and controls who received rs-fMRI and 1H-MRS, Study B consists of patients and controls who received rs-fMRI and 1H-MRS both before and after 2 days of riluzole treatment. **B** A, 1H-MRS provides a single scalar value for each subject. B, rs-fMRI data is gathered from the same subjects. C, Time series extracted from 333 cortical nodes and correlated to produce a connectivity matrix. D, Network Based Statistic used to identify functional connectivity network associated with neurochemical measure. E, This neurochemical associated network is characterised on the basis of number of edges shared with canonical resting state networks.

The experimental approach is summarised in Fig. 1. In both studies we used rs-fMRI to characterise functional brain networks, and used 1H-MRS to measure glutamate concentrations. In study A we used 1H-MRS to measure glutamate concentrations in individuals with schizophrenia and healthy controls. In study B, in a separate cohort of patients with treatment resistant schizophrenia and healthy controls, we measured glutamate concentrations and connectivity before and after the administration of the glutamatergic modulator riluzole. In both studies the relationship between glutamate concentrations and network connectivity was examined using NBS^18^. Any glutamate associated networks identified were then further characterised to determine whether they overlapped with the salience network to a greater extent compared to other canonical resting state networks (default mode, visual, frontoparietal, auditory, dorsal attention, ventral attention, and sensorimotor).

### Study A: participants

Study A contained 76 individuals meeting DSM-IV criteria for schizophrenia and 82 controls. Patients were recruited from the University of New Mexico Hospitals, and if treated were clinically stable on the same antipsychotic medication for over 4 weeks. Participants received both rs-fMRI and 1H-MRS scans during the same scanning session. The 1H-MRS data for this study has been previously reported and further details are available^19^, a network analysis integrating rs-fMRI and 1H-MRS has not previously been undertaken.

### Study A: image acquisition and preprocessing

Scans were acquired using a Siemens Trio 3 T MRI scanner. 1H-MRS was performed with a phase-encoded version of a point-resolved spectroscopy sequence both with and without water pre-saturation as previously described^20^. The following parameters were used: TE = 40 ms; TR = 1500 ms; slice thickness = 15 mm; total scan time = 582 s. The 1H-MRS volume of interest was prescribed from an axial T2 weighted image to lie immediately above the lateral ventricles and parallel to the anterior–posterior commissure axis, and included portions of the cingulate gyrus and the medial frontal and parietal lobes (although only voxels lying within the medial frontal cortex were used in subsequent analysis, see eFig. 1).

Resting state fMRI was acquired during the same session using the following parameters: TR = 2 s; TE = 29 ms; interleaved ascending acquisition; 164 time points; slice thickness 3.5 mm, slice spacing 4.55 mm; spatial positions 33, flip angle 75; matrix size 64*64; total scan time 328 s. Preprocessing was performed using fMRIPrep 20.0.5^21^, with denosing performed using the eXtensible Connectivity Pipeline (XCP) software with the ‘36pdespike’ design file which involved regressing out quadratic terms, and squares of derivatives of six motion, two physiological time series (CSF and white matter), global signal regression, and despiking of frames that exceeded a threshold of 0.5 mm FD or 1.5 standardised DVARS (spatial standard deviation of successive difference images)^22^.

Full details of both 1H-MRS and rs-fMRI methods are provide in the Supplementary Information.

### Study B: participants

Study B contained 19 individuals meeting DSM-IV criteria for schizophrenia, and meeting the minimum requirement for a diagnosis of treatment resistant schizophrenia as outlined in Treatment Response and Resistance in Psychosis (TRRIP) working group consensus guidelines^23^. Patients were recruited from outpatient services within the South London and the Maudsley NHS Foundation Trust. 17 healthy controls were also recruited. Participants received baseline imaging including rs-fMRI and 1H-MRS. Following this, over 2 days both patients and controls received four separate 50 mg doses of riluzole (2-amino-6-trifluormethoxy benzothiazole), a glutamatergic modulator that reduces synaptic release of glutamate, increases cortical glutamate metabolism, and reduces the amount of releasable presynaptic glutamate^24,25^. The same imaging measures obtained at baseline were repeated 2 days later following the pharmacological interventions. The data for this study has previously been reported^26^, a network analysis integrating rs-fMRI and 1H-MRS has not previously been undertaken.

### Study B: image acquisition and preprocessing

Scans were acquired using a General Electric 3 T MRI. 1H-MRS spectra (Point resolved Spectroscopy; TE = 30 ms; TR = 3000 ms; total scan time 360 s) were acquired using the standard GE PROBE (proton brain examination) sequence. A voxel was defined within the anterior cingulate from the midline sagittal localiser, with the centre of the 20 mm × 20 mm × 20 mm voxel placed 16 mm above the genu of corpus callosum perpendicular to the AC–PC line (eFig. 2).

Resting state fMRI was acquired during the same session with a multi-echo sequence using the following parameters: TR = 2.5 s; TE = 12, 28, 44, 70 ms; 240 time-points; slice thickness = 3 mm; slice spacing = 4 mm; spatial positions, 32; flip angle 80°; field of view 240 mm; matrix size 64 × 64; total scan time 600 s. A multi-echo specific pipeline was employed for processing and denoising as previously described in which following an independent component analysis noise components are discarded from the data^26^.

Full details of both 1H-MRS and rs-fMRI methods are provide in the Supplementary Information.

### Common methods: 1H-MRS analysis

1H-MRS Spectra were analysed using LC Model^27^. Metabolites were corrected for the proportion of CSF present in the voxel, and poor quality scans (Cramér–Rao minimum variance bounds <20%, and signal to noise ratio ≤5) were excluded from analysis. Further details are provided in the Supplementary Materials.

### Common methods: rs-fMRI analysis

Time series were extracted from the 333 nodes of the Gordon cortical atlas^28^. Functional connectivity between a pair of nodes was defined as the Pearson correlation coefficient between the nodes’ time series. For each participant a network was constructed, in which each ‘edge’ linking a pair of nodes was defined as the functional connectivity of those nodes.

### Common methods: identifying glutamate associated networks

We used the Network Based Statistic as previously described^18,29^, to investigate the relationship between glutamate concentrations and connectivity. This involves first running a linear regression at each edge in the whole brain (333 node*333 node) network where connectivity strength is the dependent variable, and the glutamate concentration the predictor variable, to create a single group-level network, in which each edge is the test statistic that represents the strength of this glutamate-connectivity association. In the next step, edges within this group-level network are discarded if they lie below a threshold, and the number of edges within the densest remaining component (i.e. the component containing the greatest number of edges) is calculated. This is then compared to a null distribution that is generated by following the same procedure, but where the group-level correlation network has been constructed from permuted glutamate measures (see Supplementary Materials for further details). The magnitude of the threshold determines the size of the network one identifies, with weaker thresholds identifying widespread diffuse relationships and more stringent thresholds identifying the core network showing the strongest relationship. We focused on core networks and investigated ten thresholds of 2.5 ≤ t ≤ 2.9, performing 5000 permutations at each threshold (see eFig. 3 for further illustration of the method).

For study A glutamate concentration was the predictor variable, and connectivity the dependent variable; for study B change in glutamate concentration was the predictor variable, and change in connectivity the dependent variable. For both analyses we first investigated whether the glutamate-connectivity relationship showed any evidence of differing between patients and controls by testing for the presence of an interaction effect. If no interaction effect was present, we tested for a glutamate-connectivity relationship in the cohort as a whole. In addition to the bivariate relationship between glutamate and connectivity we also performed analyses controlling for age and sex (see Supplementary for further details).

### Common methods: characterising glutamate associated networks

We next characterised the overlap between the glutamate associated networks identified in the previous step and the salience network. Nodes were assigned to canonical networks as specified by Gordon et al. (default mode, frontoparietal, auditory, salience, dorsal attention, sensorimotor, other)^28^. We calculated the proportion of salience network edges (any edge linking two salience network nodes) that overlapped with edges of the glutamate associated network at each NBS threshold (correcting for size of the neurochemical associated network). We also calculated the overlap with the other canonical networks. We then tested whether the overlap with the salience network was greater than expected by chance at each NBS threshold by comparing the observed overlap with the overlap obtained in 5000 null networks generated by correlating permuted glutamate measures with connectivity matrices, and thresholding to the equivalent size as the observed network. In Study A we also undertook further exploratory analyses of the glutamate associate network by examining whether its mean connectivity at the most stringent NBS threshold was associated with patient-control status or symptom severity (positive and negative syndrome scale (PANSS) total and subscale scores, and Measurement-and-Treatment-Research-to-Improve-Cognition- in-Schizophrenia (MATRICS) composite and subscale scores).

## Results

### Study A: glutamate associated networks

Study A involved 82 controls and 76 patients. Participants had a mean age of 36.7 years and were 78% male, patients had a mean PANSS score of 63.3 and 89% were receiving treatment with antipsychotics (see Table 1). All subjects had at least one valid 1H-MRS measurement of frontal cortex glutamate concentrations, and Cramér Rao lower bounds ranged from 14.0 to 17.7 (mean = 15.7, *SD* = 0.82).

**Table 1:** Demographic details of study participants.

	Study A			Study B		
	Control	Patient	*p*	Control	Patient	*p*
*N*	82	76		17	19	
Age, mean (*SD*)	38.0 (12.2)	35.4 (13.9)	0.22	37.0 (8.9)	39.7 (10.9)	0.43
Sex (% male)	67%	89%	0.09	88%	84%	0.89
PANSS Positive mean (*SD*)	NA	16.4 (6.1)		NA	18.5 (3.0)	
PANSS Negative mean (*SD*)	NA	16.5 (6.2)		NA	19.6 (5.5)	
PANSS General mean (*SD*)	NA	30.3 (11.2)		NA	34.4 (8.0)	
PANSS Total mean (*SD*)	NA	63.3 (19.9)		NA	72.5 (10.2)	
MATRICS overall	50.0 (9.6)	29.2 (13.3)	<0.001	NA	NA	
Antipsychotic treated (%)	NA	89%		NA	100%	
Antipsychotic dose (olanzapine equivalents/mg), mean (*SD*)	NA	13.3 (12.9)		NA	16.8 (9.8)	

*MATRICS* measurement and treatment research to improve cognition in schizophrenia, *PANSS* positive and negative syndrome scale.

**Fig. 2:**
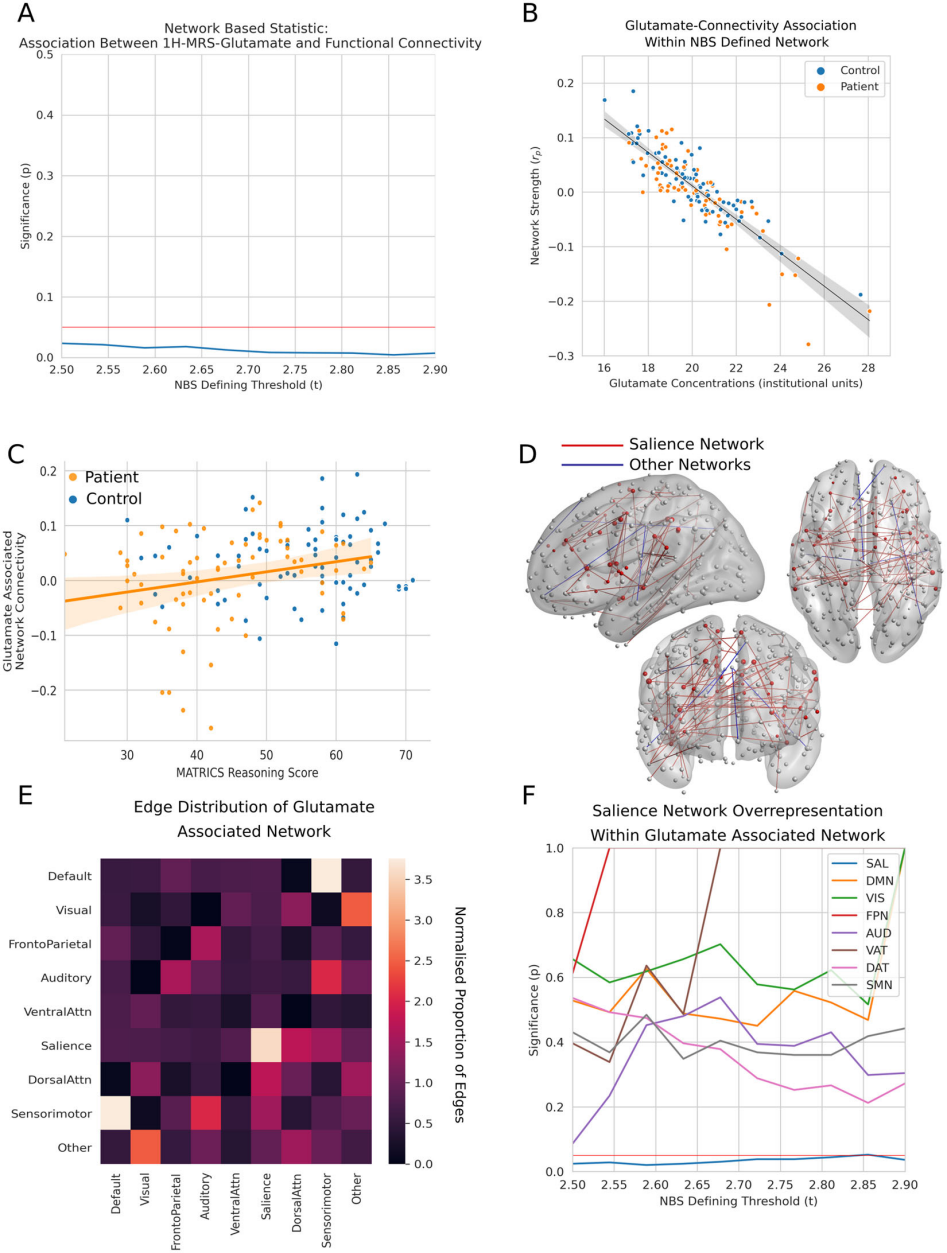
Study A: glutamate associated networks. **A** Higher anterior cingulate glutamate concentrations are associated with reduced connectivity across a range of NBS thresholds. The horizontal red line represents the *p* < 0.05 threshold while the blue line represents statistical significance across a range of thresholds. **B** Illustration of glutamate-connectivity relationship at a single threshold. Mean connectivity within the glutamate associated network for the NBS threshold of *t* = 2.5 is negatively associated with glutamate concentrations. **C** Mean connectivity within the glutamate associated network was positively associated with MATRICS reasoning scores in patients (*r*_s_ = 0.25, *p* = 0.04, *df* = 75), and the sample as a whole (*r*_*s*_ = 0.17, *p* = 0.04, *df* = 157). **D** The glutamate associated network for the threshold *t* = 2.5. Only intra canonical network edges are shown, intra salience network edges are show in red, while all other networks are shown in blue. A relative excess of salience network edges is apparent. **E** Heatmap illustrating the proportion of canonical intra- (diagonal) and inter-(off diagonal) network edges that overlap with the glutamate associated network. A normalised proportion of edges greater than one indicates a relative excess of edges compared to what would be expected to an even distribution of edges across the cortex. Salience network edges show greater overlap with the glutamate associated network than other canonical networks. **F** Results of permutation testing show that the excess of salience overlapping edges within the glutamate associated network is statistically significant across a range of NBS thresholds. The horizontal red line represents a threshold of *p* < 0.05, while the other lines represent the statistical significance of the overlap between a canonical network and the glutamate associated network. Only the salience network is significantly overrepresented, and this is at all thresholds. *AUD* auditory, *DAT* dorsal attention, *DMN* default mode, *FPN* frontoparietal, *SAL* salience, *SMN* Sensorimotor, *VAT* ventral attention, *VIS* visual.

When examining the relationship between glutamate concentrations and connectivity a network in which glutamate concentrations were negatively associated with connectivity was identified across a wide range of NBS thresholds (Fig. 2). There was no evidence of an interaction between patients and controls (*N* = 158, *p* > 0.84 all thresholds), which indicates that a similar relationship between glutamate concentrations and connectivity is observed in both patients and controls. This network ranged in size from 188 edges (0.098% of the fully connected 55,278 whole brain network) at the most stringent threshold, to 539 edges (0.34% of the whole brain network) at the weakest threshold. When controlling for age and sex this relationship was not significantly altered (eFig. 4).

**Fig. 3:**
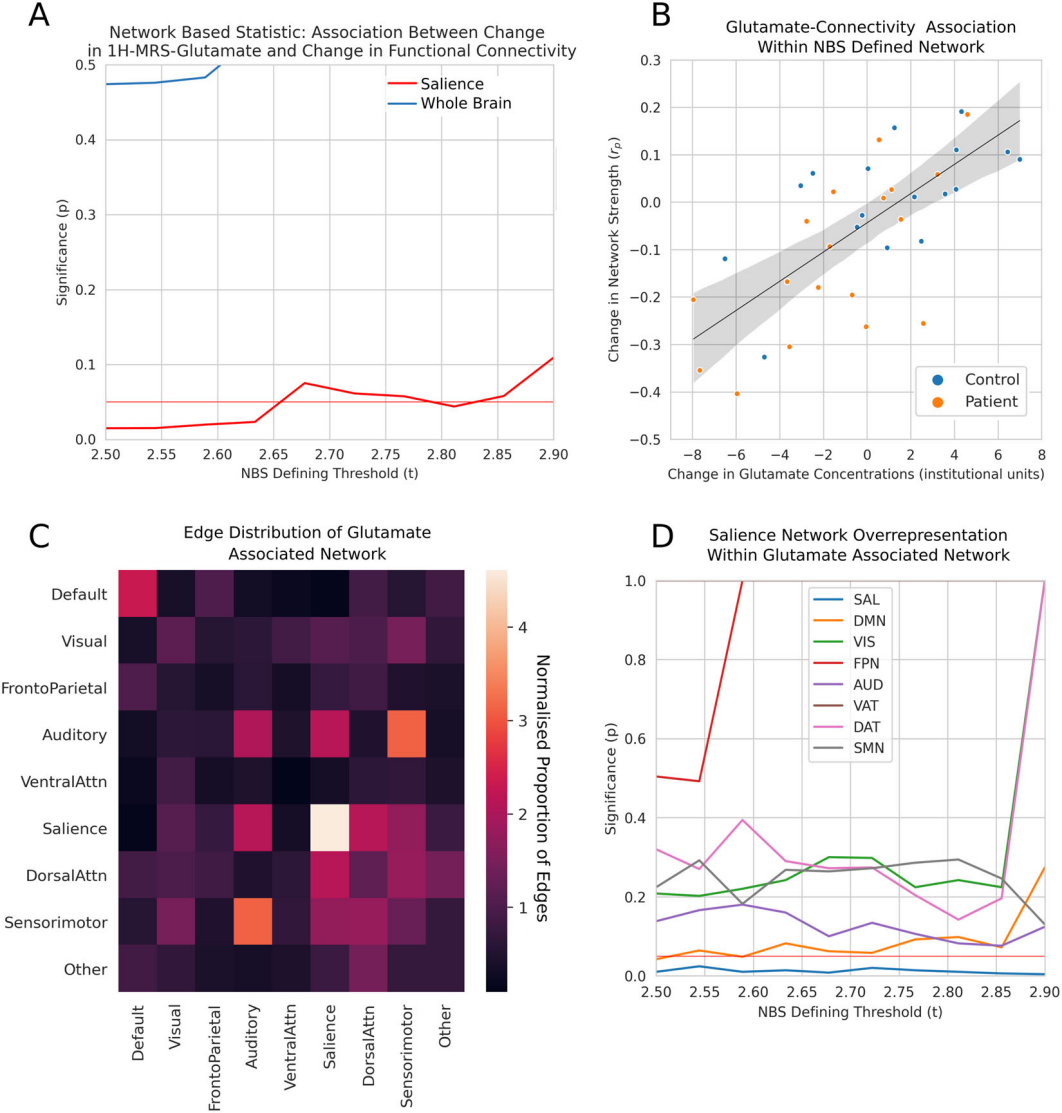
Study B: riluzole associated change in glutamate concentrations and network connectivity. **A** When analysis is constrained to the salience network, at certain NBS thresholds there is a positive association between change in glutamate concentrations and change in connectivity following riluzole administration. The horizontal red line represents the *p* < 0.05 threshold, the other red line represents statistical significance when analysis is constrained to the salience network, while the blue line represents statistical significance for the whole brain analysis. **B** Illustration of the change in glutamate—change in connectivity relationship at a single threshold. Change in mean connectivity within the glutamate associated network for the NBS threshold of *t* = 2.5 is positively associated with the change in glutamate concentrations. **C** Heatmap illustrating the proportion of canonical intra- (diagonal) and inter-(off diagonal) network edges that overlap with the glutamate associated network. A normalised proportion of edges greater than one indicates a relative excess of edges compared to what would be expected to a even distribution of edges across the cortex. Salience network edges show greater overlap with the glutamate associated network than other canonical networks. **D** Results of permutation testing show that the excess of salience overlapping edges within the whole brain change in glutamate associated network is statistically significant across a range of NBS thresholds. The horizontal red line represents a threshold of *p* < 0.05, while the other lines represent the statistical significance of the overlap between a canonical network and the glutamate associated network. Only the salience network is significantly overrepresented, and this is at all thresholds. *AUD* auditory, *DAT* dorsal attention, *DMN* default mode, *FPN* frontoparietal, *SAL* salience, *SMN* sensorimotor, *VAT* ventral attention, *VIS* visual.

The edges of the glutamate associated network were disproportionately located within the salience network (Fig. 3A), and this was statistically significant at a wide range of NBS thresholds with no evidence of overrepresentation of any other network (Fig. 3A, B).

Mean connectivity with the glutamate associated network was weaker in individuals with schizophrenia (*t* = 2.23, *p* = 0.027, *df* = 156). Mean connectivity within the glutamate associated network was not significantly associated with severity of symptoms as defined by the PANSS (Positive *r*_s_ = −0.09, *p* = 0.46, Negative *r*_s_ = 0.10, *p* = 0.41, General *r*_s_ = −0.07, *p* = 0.52, Total *r*_s_ = 0.03, *p* = 0.8; *df* = 75). When examining the association with cognitive symptoms in the sample as a whole, both processing speed (*r*_s_ = 0.18, *p* = 0.03, *df* = 157) and reasoning (*r*_s_ = 0.17, *p* = 0.04, *df* = 157) subdomains were significantly associated with mean connectivity of the glutamate associated network. In patients alone only reasoning (*r*_s_ = 0.25, *p* = 0.04, *df* = 75, see Fig. 2C) showed a significant association, and no significant associations were observed in controls. None of these associations survive correction for multiple comparisons.

### Study B: glutamatergic network modulation

Study B involved 17 controls and 19 patients, participants had a mean age of 38.4 years and were 86% male, patients had a mean PANSS score of 72.5 and all were receiving antipsychotic treatment (see Table 1). Cramér Rao lower bounds ranged from 4.0–12.0 (mean = 6.53, *SD* = 1.44).

When examining the relationship between change in glutamate concentrations and change in connectivity there was no evidence of an interaction between patients and controls (*N* = 36, *p* > 0.4 all thresholds). The whole brain analysis identified networks representing an association between change in glutamate concentrations and change in connectivity, ranging in size from 63 edges (0.011% of the fully connected 55,278 whole brain network) at the most stringent threshold, to 481 edges (0.87% of the whole brain network) at the weakest threshold, but this was not statistically significant at any thresholds (Fig. 3A). When analysis was restricted to the salience network (in keeping with previous approaches^29^) there was again no interaction seen between patient and control groups (*N* = 36, *p* > 0.4 all thresholds), but a significant positive association was seen between change in glutamate concentrations and change in network connectivity (Fig. 3A) at certain NBS thresholds, and when age and sex were controlled for this was significant at all NBS thresholds (eFig. 5).

When examining the whole brain network described in the above paragraph (i.e. the network illustrated by the blue line in Fig. 3A consisting of connections most strongly associated with glutamate concentrations), we found that this was still disproportionately composed of edges located within the salience network, and this was statistically significant at a wide range of NBS thresholds with no evidence of overrepresentation of any other network (*p* < 0.05 all thresholds, Fig. 3C, D).

The results for both studies at a wider range of NBS thresholds are illustrated in eFig. 6 and 7.

## Discussion

We have demonstrated in a large sample of individuals with schizophrenia and healthy controls that glutamate concentrations in the frontal cortex are associated with the connectivity within the salience network. This glutamate associated network was weaker in patients compared to controls, and in an exploratory analysis connectivity within the network showed some associations with cognitive symptoms. Furthermore, pharmacologically induced changes in frontal cortex glutamate concentrations were also associated with connectivity patterns that localised to the salience network. The fact that glutamate function, long thought to be relevant to schizophrenia pathophysiology, is linked to salience network connectivity adds further weight to the hypothesis that this network plays a fundamental role in schizophrenia^9^. Furthermore, these findings have a general transdiagnostic relevance given the fact that disruption of the salience network, and altered glutamate signalling is also seen in other psychiatric illnesses such as affective and substance use disorders^7,8,30,31^.

Several recent studies have investigated the relationship between glutamate concentrations and resting state networks^32–38^. These studies, however, have generally consisted of relatively small samples, and so have been unable to take a whole brain approach, using instead a small number of prespecified seeds. Due to our larger sample size we were able to parcellate the entire cortex into 333 nodes with no a priori preference being given to any particular regions. This unbiased approach allowed for subsequent inferences that allowed us to identify the salience network as being specifically associated in both studies.

Our finding that glutamate concentrations have a negative relationship with salience network connectivity complements our previous finding that striatal dopamine synthesis capacity has a positive relationship with connectivity of the same network^29^. These two findings are also consistent with work that has highlighted negative associations between anterior cingulate glutamate concentrations and striatal dopamine synthesis capacity^29,39,40^. The lack of a significant interaction by group suggests that while both glutamate concentrations and functional connectivity may both be individually altered in schizophrenia, the relationship between the two is not clearly disrupted.

A recent study found that NMDA antagonism decreases salience network connectivity^41^, which is in keeping with our finding of a negative association between glutamate concentrations and connectivity if higher glutamate concentrations are taken to represent a response to relatively reduced NMDA signalling. The analysis of change in glutamate and change in connectivity in Study B showed increases in glutamate concentrations positively associated with increases in salience network connectivity, this was in the opposite direction to the negative association observed cross-sectionally in study A. This discrepancy in the direction of the relationship highlights that observations at a between individual level do not necessarily reflect mechanisms occurring within individuals. Our previous analysis examining the effects of riluzole used a seed-to-voxel approach to investigate the relationship between change in glutamate concentrations and change in connectivity between anterior cingulate cortex and anterior prefrontal cortex^26^. The finding of a negative glutamate-connectivity association in this original analysis is not inconsistent with the current finding of a positive association as the present analysis takes a cortex wide approach, but this does not preclude the existence of individual connections showing an opposing relationship.

The current findings have clear relevance for therapeutic interventions, suggesting that modulation of glutamate signalling may have the potential to bring about changes in network connectivity. The fact that glutamate-connectivity relationships did not differ significantly between patients and controls suggests that results of studies in healthy controls may be readily translatable to patient populations.

### Limitations and future directions

The use of two separate cohorts at different sites, using different scanners, may complicate between study comparisons. However, the fact that both studies independently identified networks centred upon the salience network, despite differences in image acquisition and preprocessing, strengthens confidence in the validity of this observation. We did not find differences between patients and controls in terms of glutamate-connectivity associations, and it is possible that confounding variables masked a difference here. While groups were balanced for age and sex, and results were unchanged when controlling for these variables, it was not possible to examine the influence of antipsychotic medication given that the great majority of patients were receiving treatment. Future work should examine the relationship in antipsychotic naïve cohorts.

^1^H-MRS provides an imprecise index of the glutamate system, and is unable to distinguish between intra- and extra-cellular compartments. PET has the potential to more accurately characterise which aspects of glutamate signalling are linked to functional connectivity, but no reliable ligands currently exist. GluCest imaging may also provide further insights due to its greater spatial resolution and ability to provide whole brain coverage, however, its specificity for glutamate has been recently questioned^42^.

However, even improved characterisation of glutamatergic function, cannot directly elucidate the mechanisms via which changes in synaptic transmission propagate to macroscale changes in cortical connectivity. When considering the association between measures of glutamate concentrations and those of functional connectivity there is a question as to causal primacy. If one views functional connectivity as representing neuronal dynamics interacting via a structural connectome, however, then the two measures are intertwined and it is possible for both to have a causal role as part of a complex system. Further clinically relevant questions remain about whether modulation of either glutamate or connectivity has the potential to normalise the wider system as a whole. The use of preclinical methods to dissect molecular mechanisms, combined with biophysically informed models to bridge the gap between microscale processes and macroscale neural recordings may have the potential to advance mechanistic understanding^43^.

## Conclusions

This work provides evidence that links frontal cortex glutamate concentrations to the functional connectivity of the salience network in individuals with schizophrenia and healthy controls. The findings suggest that neurochemical modulation of glutamate signalling may have the potential to address connectivity disturbances in psychosis, although the data from the pharmacological challenge illustrates that this is not straightforward. A fuller understanding may be brought about by work focused on dissecting the precise mechanisms underlying the relationship between glutamate function and macroscale connectivity.

## Supplementary information

Supplementary
