# Supplementary material for: Glutamate connectivity associations converge upon the salience network in schizophrenia and healthy controls
Source: Transl Psychiatry. 2021 May 27;11:322. doi: 10.1038/s41398-021-01455-y (PMC8159959; doi:10.1038/s41398-021-01455-y)
Supplement: Supplementary file 1 — Supplementary [file 41398_2021_1455_MOESM1_ESM.docx]

**Glutamate connectivity associations converge upon the**

**the salience network in schizophrenia and healthy controls**

McCutcheon et al.

Supplementary information

[Methods 2](#_Toc71038423)

[1. Participants 2](#_Toc71038424)

[1.1 Study A 2](#_Toc71038425)

[1.2 Study B 2](#_Toc71038426)

[2. Image Acquisition 4](#_Toc71038427)

[2.1 1H-MRS acquisition 4](#_Toc71038428)

[2.2 Resting State Acquisition 6](#_Toc71038429)

[3. Image Analysis 7](#_Toc71038430)

[3.1 1H-MRS analysis 7](#_Toc71038431)

[eFigure 1. Study A 1H-MRS acquisition 8](#_Toc71038432)

[eFigure 2. Study B 1H-MRS acquisition 10](#_Toc71038433)

[3.2 fMRI Preprocessing 11](#_Toc71038434)

[3.3 Connectivity Analysis 14](#_Toc71038435)

[eFigure3. Description of the Network Based Statistic 15](#_Toc71038436)

[4. Results 16](#_Toc71038437)

[eFigure 4. Study A association between glutamate and connectivity after controlling for age and sex 16](#_Toc71038438)

[eFigure 5. Study B association between change in glutamate and change in connectivity after controlling for age and sex 16](#_Toc71038439)

[eFigure 6. Study A: Glutamate associated networks at wider range of NBS thresholds 17](#_Toc71038440)

[eFigure 7. Study B: Glutamate associated networks at wider range of NBS thresholds 17](#_Toc71038441)

[References 19](#_Toc71038442)

# Methods

## 1. Participants

### 1.1 Study A

Participants with schizophrenia were recruited from the University of New Mexico Hospitals. Inclusion criteria consisted of: (1) A diagnosis of DSM-IV-TR schizophrenia made through consensus by 2 research psychiatrists using the SCID-DSM-IV-TR; (2) if treated, participants were clinically stable on the same antipsychotic medications for >4 weeks. Exclusion criteria consisted of a diagnosis of neurological or current substance use disorder (except nicotine). Healthy controls exclusion criteria consisted of: (1) any DSM-IV-TR axis-I disorder (SCID-DSM-IV-TR Non-Patient-Version); (2) first-degree relatives with psychotic disorders; (3) history of neurological disorder. The study was approved by the University of New Mexico Institutional Review Board. Subjects gave written informed consent.

### 1.2 Study B

Participants meeting DSM-IV criteria for schizophrenia were recruited from outpatient services within the South London and the Maudsley NHS Foundation Trust.^1^ Healthy volunteers with no history of psychiatric illness were recruited from the local population. Exclusion criteria for all participants were as follows: inability to provide written informed consent; co-morbid drug or alcohol abuse/ dependence; a history of liver disease or transaminitis > 2 times the upper limit of normal (owing to the potential for riluzole to cause liver dysfunction); any contraindication to MRI scanning at 3 T (e.g. metallic implants); any comorbidity that could compromise scanning safety (e.g. severe asthma); pregnancy/breast feeding; and the use of medication with recognised effect on glutamatergic signalling, including clozapine, lamotrigine, lithium, carbamazepine, opiates, and psychostimulants.

﻿Treatment-resistant schizophrenia was defined as presence of at least one positive and one negative symptom rated as ≥ 4 on the PANSS, indicative of at least moderate severity, and a score of< 60 on the Global Assessment of Functioning scale (GAF) indicative of at least moderate functional impairment,^2^ despite 2 trials of an antipsychotic. A sufficient antipsychotic trial was defined as one given for at least 6 weeks with evidence of concordance (based on examination of patient records) and at a target dose recommended by the relevant manufacturer’s summary of product characteristics/at a total daily dose equivalent to or greater than 600 mg chlorpromazine. Patients were required to be on a stable antipsychotic regimen, with no change in treatment dose in the 6 weeks prior to study participation. Antipsychotic plasma levels were measured to assess concordance, as previously described.^3^ This approach to defining treatment-resistant schizophrenia conformed with at least the minimum requirements provided by Treatment Response and Resistance in Psychosis (TRRIP) working group consensus guidelines.^4^

## 2. Image Acquisition

### 2.1 1H-MRS acquisition

*Study A*

﻿Scans were acquired using a Siemens Trio 3T MRI scanner (VB-17; 12 channel head-coil). T1-weighted structural scans were obtained with 3D-MPRAGE for voxel tissue segmentation (TR=1500ms, TE=3.87ms). 1H-MRS was performed with a phase-encoded version of a point-resolved spectroscopy sequence both with and without water pre-saturation as previously described.^5^ The following parameters were used: TE = 40 ms, TR = 1500 ms, slice thickness = 15 mm, FOV = 220 × 220 mm, circular k-space sampling (radius = 12), Cartesian k-space size = 32 × 32 after zero filling, k-space Hamming filter with 0.5 width and number of averages = 1, total scan time = 582 seconds. The nominal voxel size was 0.71 cm^3^ but the effective voxel volume was 2.4 cm^3^. The 1H-MRS volume of interest was prescribed from an axial T2 weighted image to lie immediately above the lateral ventricles and parallel to the anterior–posterior commissure axis, and included portions of the cingulate gyrus and the medial frontal and parietal lobes. To minimize the chemical shift artifact, the transmitter was set to the frequency of the NAA methyl-peak during the acquisition of the metabolite spectra and to the frequency of the ﻿water-peak during the acquisition of the unsuppressed water spectra. Additionally, the outermost rows and columns of the VOI were excluded from analysis.

*Study B*

*﻿*Scans were acquired using a General Electric (Milwaukee, WI, USA) 3T MRI. Each scanning session commenced with a localizer, standard axial T2-weighted fast spin echo scan and a T1-weighted structural scan. The T1-weighted image (﻿TR/TE = 7.312/3.01 ) was used to plan 1H-MRS voxel placement and for voxel segmentation. The 1H- MRS voxel was placed in the ACC. The ACC voxel was defined from the midline sagittal localizer, with the centre of the 20 mm × 20 mm × 20 mm voxel placed 16 mm above the genu of corpus callosum perpendicular to the AC–PC line (eFigure. 2). 1H-MRS spectra (Point RESolved Spectroscopy; TE = 30 ms; TR = 3000 ms; 96 averages; bandwidth = 5 kHz, number of data points = 4096) were acquired using the standard GE PROBE (proton brain examination) sequence. Additional unsuppressed water reference spectra (16 averages) were acquired for eddy current correction and water scaling.

### 2.2 Resting State Acquisition

*Study A*

Rs-fMRI data was obtained using the same Siemens Trio scanner 3T as for 1H-MRS. During the scan, participants were instructed to keep their eyes open and look at a fixation cross. MRI *﻿*Resting state fMRI was acquired using the following parameters: TR=2s; TE = 29ms; interleaved ascending acquisition; 164 time points; slice thickness 3.5mm, slice spacing 4.55mm; spatial positions 33, flip angle 75; matrix size 64*64.

*Study B*

﻿Rs-fMRI data was obtained using the same General Electric (Milwaukee, WI, USA) 3T as for 1H-MRS. During the scan, participants were instructed to keep their eyes open and look at a fixation cross. MRI *﻿*Resting state fMRI was acquired using a multi-echo echo planar imaging (ME-EPI) sequence: TR= 2.5 s; TE = 12, 28, 44, 70 ms; 240 time-points; slice thickness = 3mm; slice spacing = 4 mm; spatial positions, 32; flip angle 80°; field of view 240 mm; matrix size 64 × 64.

## 3. Image Analysis

### 3.1 1H-MRS analysis

*Study A*

﻿Data was automatically preprocessed and fit using LCModel (Version 6.11). Voxels of poor quality as defined by poorly fitted metabolite peaks (Cramér–Rao minimum variance bounds > 20%, and signal to noise ratio ≤ 5, as reported by LCModel) were excluded from further analysis. ﻿The results from LCModel for the metabolites were corrected for partial volume (using SPM-5 segmented T1 images) and relaxation effects (from literature values), and estimated as concentrations in millimoles per kg of 1H-MRS visible tissue water (mM).^6^ In order to minimize bias by small errors of cerebro-spinal fluid (CSF) segmentation, voxels were classified as “predominantly gray” (100*GM/GM+WM > 66%), “predominantly white” (100*GM/GM+WM < 34%) or mixed (the remaining voxels). Finally, regions-of-interest (ROIs) were selected from “predominantly” GM and WM voxels in each hemisphere, anterior (frontal) and posterior (parietal) to the central sulcus. This resulted in 6 ROI’s: frontal medial GM, left and right frontal WM, parietal medial GM, left and right parietal WM. Results from all ROIs have been reported previously but for the current analysis we further investigated solely the ‘frontal medial GM’ voxels as this most closely matched the ROI used in Study B. For each subject the glutamate concentrations were meaned across all eligible voxels (i.e. those classified as of sufficient quality, and located within the ‘frontal medial GM’ ROI) to give a single value for that subject.


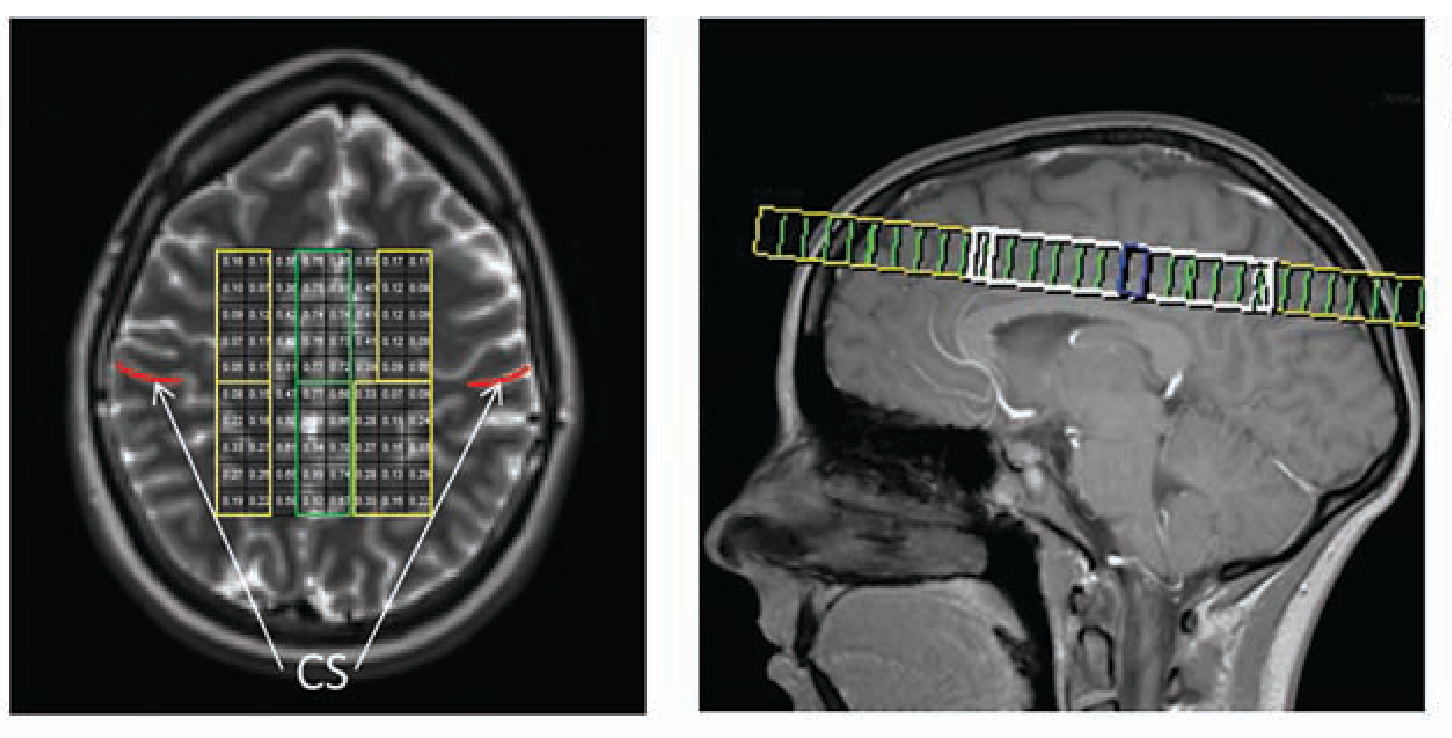

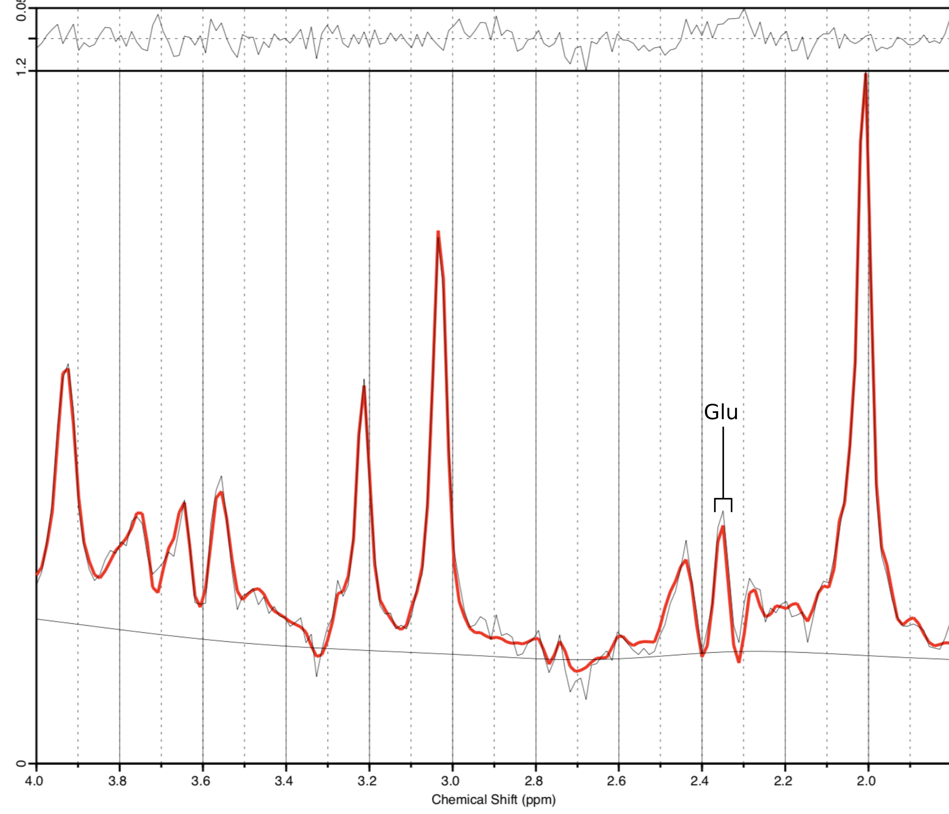


### eFigure 1. Study A 1H-MRS acquisition

Example slab placement (above) for a Study A participant. Only voxels lying within the medial frontal cortex were examined in subsequent analyses, a sample spectra from one of these voxels is shown.

*Study B*

Spectra were analysed using LC Model version 6.3-1L.^7^ Voxel grey matter (GM), white matter (WM), and cerebrospinal fluid (CSF) content for each subject were derived by extracting the location of the voxel from the spectra file headers and using an in-house script to calculate the percentage of GM, WM, and CSF using the segmented T1-weighted images. Segmentation was performed using the ‘segment’ function of SPM12. The results from LCModel for the metabolites were corrected for partial volume (using SPM-5 segmented T1 images) and relaxation effects (from literature values), and estimated as concentrations in millimoles per kg of 1H-MRS visible tissue water (mM).^6^ Poor-quality scans, as defined by poorly fitted metabolite peaks (Cramér–Rao minimum variance bounds > 20%, and signal to noise ratio < 5, as reported by LCModel) were excluded from further analysis


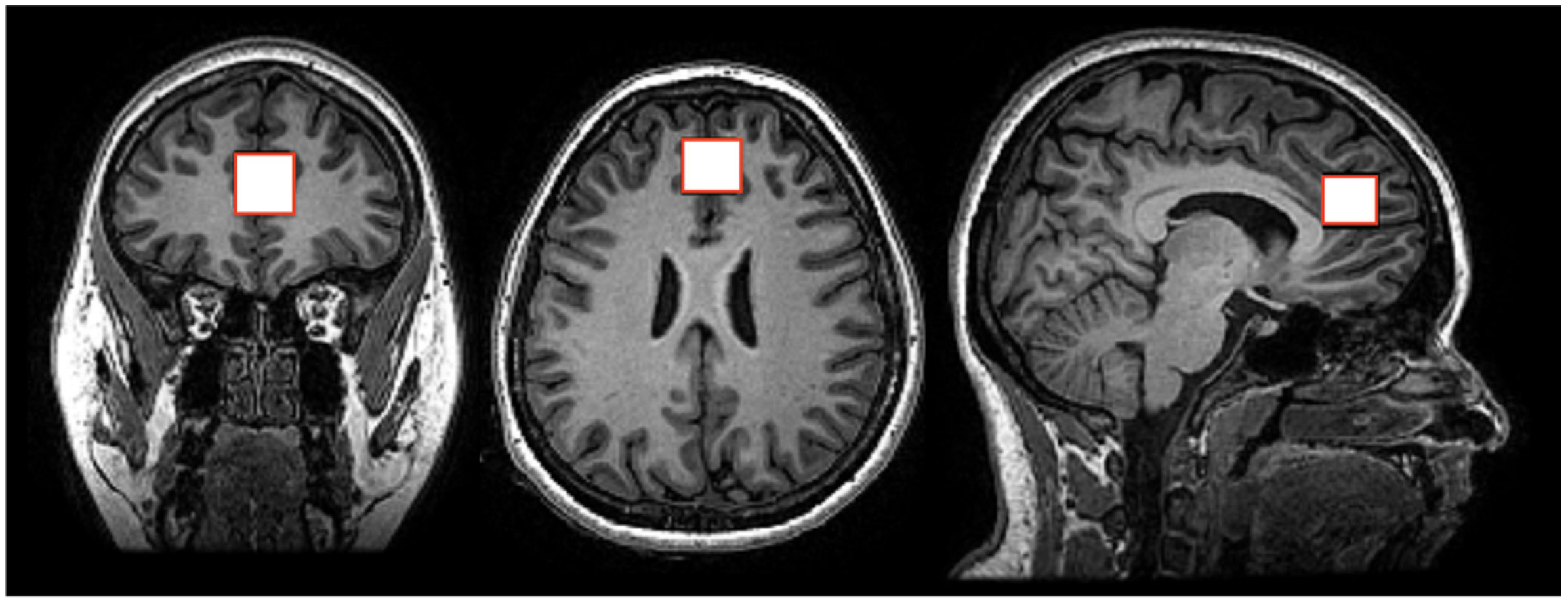



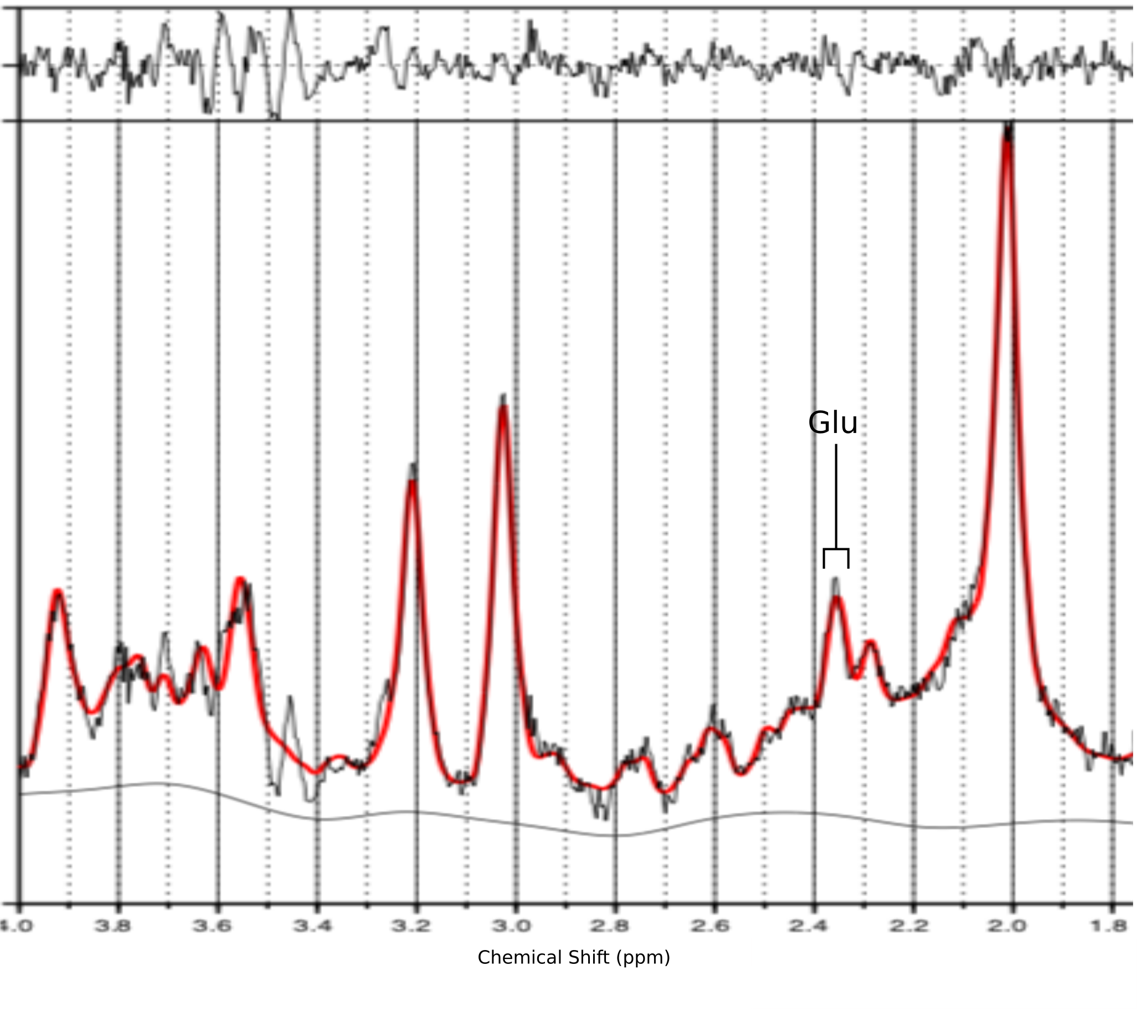


### eFigure 2. Study B 1H-MRS acquisition

Example voxel placement (above) and spectra (below) for a Study B participant

### 3.2 fMRI Preprocessing

*Study A*

Preprocessing was performed using fMRIPrep 20.0.5,^8^ which is based on Nipype 1.4.2.^9^ Anatomical data was processed first, and the T1-weighted (T1w) image was corrected for intensity non-uniformity (INU) with N4BiasFieldCorrection distributed with ANTs 2.2.0,^10,11^ and used as T1w-reference throughout the workflow. The T1w-reference was then skull-stripped with a Nipype implementation of the antsBrainExtraction.sh workflow (from ANTs), using OASIS30ANTs as target template. Brain tissue segmentation of cerebrospinal fluid (CSF), white-matter (WM) and gray-matter (GM) was performed on the brain-extracted T1w using fast.^12^ Brain surfaces were reconstructed using recon-all (FreeSurfer 6.0.1),^13^ and the brain mask estimated previously was refined with a custom variation of the method to reconcile ANTs-derived and FreeSurfer-derived segmentations of the cortical gray-matter of Mindboggle.^14^ Volume-based spatial normalization to two standard spaces (MNI152NLin6Asym, MNI152NLin2009cAsym) was performed through nonlinear registration with antsRegistration (ANTs 2.2.0), using brain-extracted versions of both T1w reference and the T1w template. FSL’s MNI ICBM 152 non-linear 6th Generation Asymmetric Average Brain Stereotaxic Registration Model used for spatial normalization.

Functional data preprocessing was then performed. First, a reference volume and its skull-stripped version were generated using a custom methodology of fMRIPrep. A deformation field to correct for susceptibility distortions was estimated based on fMRIPre’s fieldmap-less approach. The deformation field is that resulting from co-registering the BOLD reference to the same-subject T1w-reference with its intensity inverted.^15^ Registration is performed with antsRegistration (ANTs 2.2.0), and the process regularized by constraining deformation to be nonzero only along the phase-encoding direction, and modulated with an average fieldmap template.^16^ Based on the estimated susceptibility distortion, a corrected EPI (echo-planar imaging) reference was calculated for a more accurate co-registration with the anatomical reference. The BOLD reference was then co-registered to the T1w reference using bbregister (FreeSurfer) which implements boundary-based registration.^17^ Co-registration was configured with six degrees of freedom. Head-motion parameters with respect to the BOLD reference (transformation matrices, and six corresponding rotation and translation parameters) are estimated before any spatiotemporal filtering using mcflirt.^18^ BOLD runs were slice-time corrected using 3dTshift from AFNI 20160207.^19^ The BOLD time-series were resampled onto the following surfaces (FreeSurfer reconstruction nomenclature): fsaverage. The BOLD time-series (including slice-timing correction when applied) were resampled onto their original, native space by applying a single, composite transform to correct for head-motion and susceptibility distortions. These resampled BOLD time-series will be referred to as preprocessed BOLD in original space, or just preprocessed BOLD. Grayordinates files containing 91k samples were also generated using the highest-resolution fsaverage as intermediate standardized surface space. All resamplings can be performed with a single interpolation step by composing all the pertinent transformations (i.e. head-motion transform matrices, susceptibility distortion correction when available, and co-registrations to anatomical and output spaces). Gridded (volumetric) resamplings were performed using antsApplyTransforms (ANTs), configured with Lanczos interpolation to minimize the smoothing effects of other kernels.^20^ Non-gridded (surface) resamplings were performed using mri_vol2surf (FreeSurfer). Many internal operations of fMRIPrep use Nilearn 0.6.2 ,^21^ mostly within the functional processing workflow. For more details of the pipeline, see the section corresponding to workflows in fMRIPrep’s documentation.

Following fmriprep preprocessing denoising of the data in native (surface based) space was performed using ﻿eXtensible Connectivity Pipeline (XCP) software with the ‘36pdespike’ design file.^22^ This involved regressing out quadratic terms, and squares of derivatives of six motion, two physiological time series (CSF and white matter), global signal regression, and despiking of frames that exceeded a threshold of 0.5 mm FD or 1.5 standardised DVARS. Data were temporally bandpass filtered (0.008‐0.09 Hz).

For extraction of node time courses the surface based version of the Gordon atlas was resampled to fsaverage space, and the denoised fMRI data in native space was also transformed to fsaverage space using the transforms calculate by fmriprep.

*Study B*

A multi-echo specific pipeline was employed for processing and denoising of Study B data as previously described.^23^ After realignment and slice timing correction, multi-echo independent component analysis was used to denoise the resting state data.^24^ After performing an independent component analysis on the unprocessed resting fMRI data, the dependence of each component on TE is quantified. Genuine BOLD T2* signal is linearly related to TE, whereas artefactual signal is not. As a result, it is then possible to separate resting state networks from noise components. The time courses from the non-BOLD components are then used as regressors for data cleaning, along with white matter and CSF time courses. Temporal band bass filtering was performed using FSL with sigma = 50. Normalisation to MNI space was then performed using the CONN toolbox (version 17.b)13 for Statistical Parametric Mapping software (SPM 12 (6906)).^25^ Node time courses were then extracted from the volumetric version of the Gordon atlas.

### 3.3 Connectivity Analysis

Time‐series were extracted from N=333 predefined nodes of interests of the Gordon cortical atlas. For each participant, a graph representing a functional connectivity network was constructed, each edge representing the level of functional connectivity between a pair of nodes, which was computed as the Pearson’s correlation coefficient between their mean time‐series.

*Network Based Statistic*

The network based statistic (NBS) relies on permutation testing to test statistical significance. eFigure3 describes the method. This employs the method of Freedman & Lane to perform permutation testing with the general linear model.^26^ For full details on how nuisance variables are accounted for in this framework please see the NBS manual.^27^

*Canonical Networks*

Nodes were assigned to networks based on the original labelling of Gordon et al.^28^ Only networks with at least ten nodes were included. The ‘sensorimotor mouth’ and ‘sensorimotor hand’ networks were combined to make a single ‘sensorimotor’ network, and ‘salience’ and ‘cinguloopercular’ were combined to make a single ‘salience’ network.


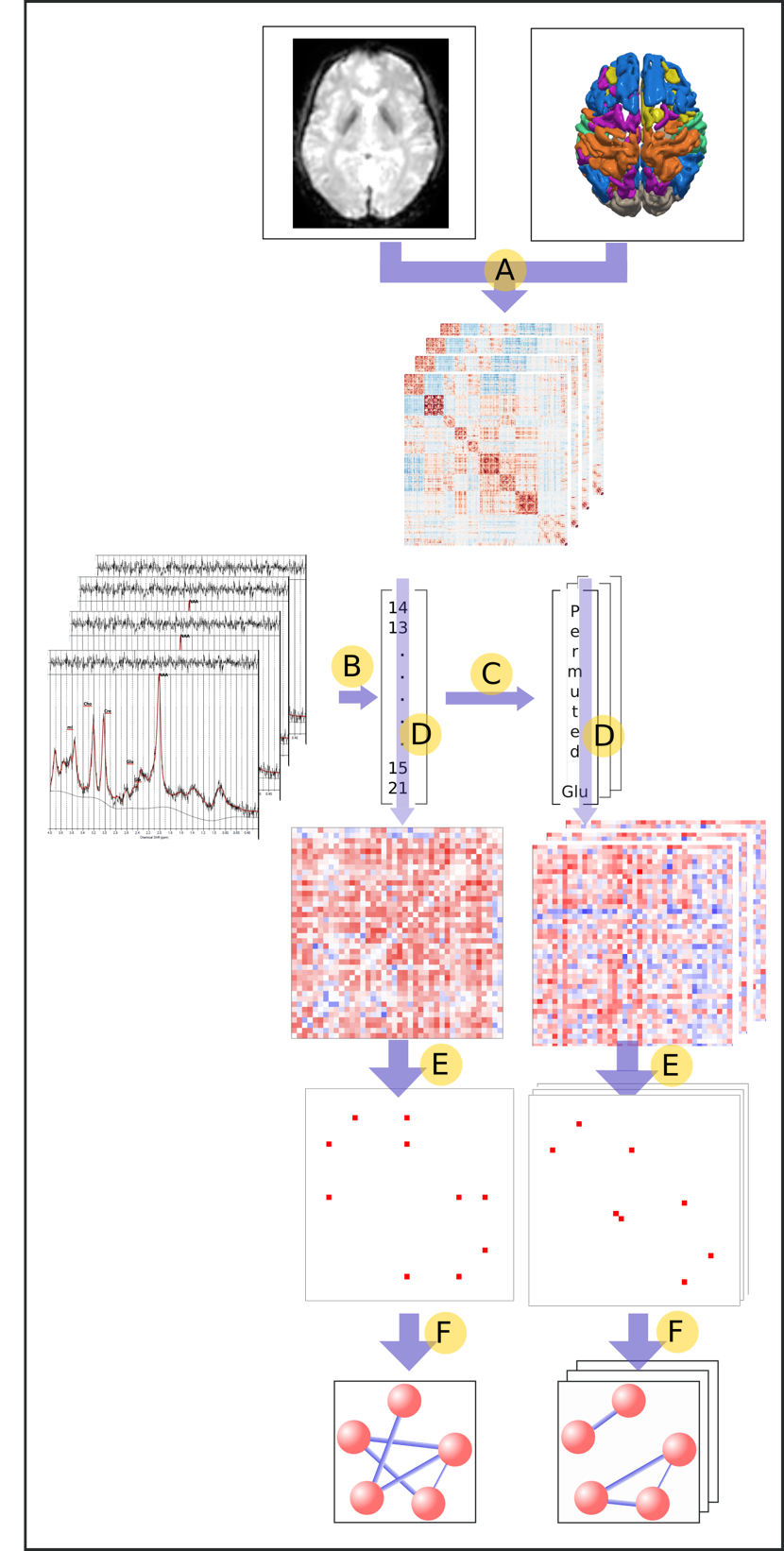


### eFigure3. Description of the Network Based Statistic

1. Individual functional connectivity networks constructed from rs-fMRI data for each participant using the Gordon parcellation.
2. Frontal cortex glutamate concentration calculated for each participant
3. Glutamate concentrations randomly permuted across participants 5,000 times
4. Group level glutamate associated network constructed where each edge represents the correlation between that edge’s functional connectivity and the glutamate values.
5. The glutamate associated network is thresholded and binarized
6. The number of edges of the largest connected component in the observed glutamate associated network (5 in the depicted example) is compared to the permuted graphs (3 edges displayed in the example). P values are calculated based on the proportion of permuted example the observed example is greater than.

# 4. Results


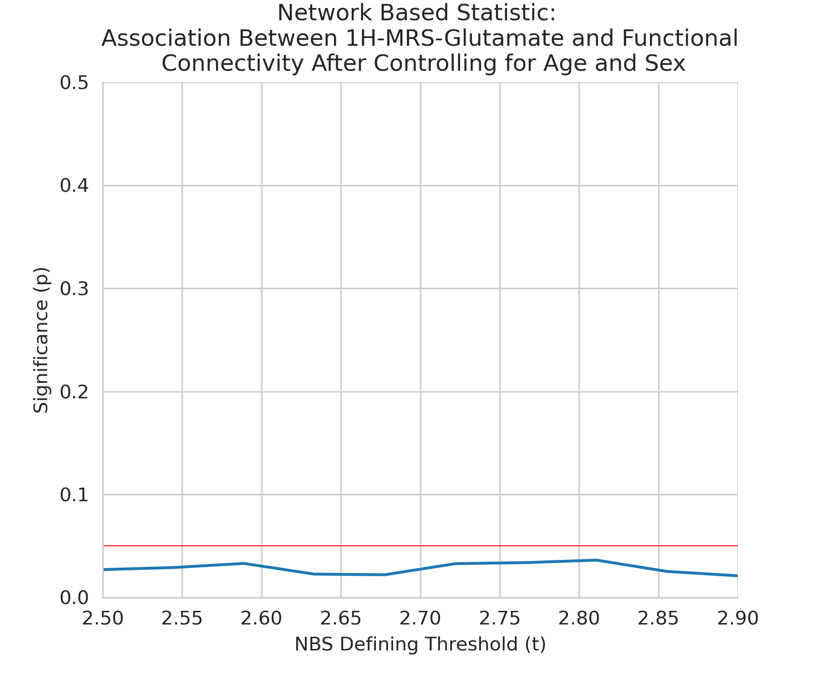


### eFigure 4. Study A association between glutamate and connectivity after controlling for age and sex

A network in which functional connectivity is negatively associated with frontal cortex glutamate concentrations is observed after controlling for age and sex.


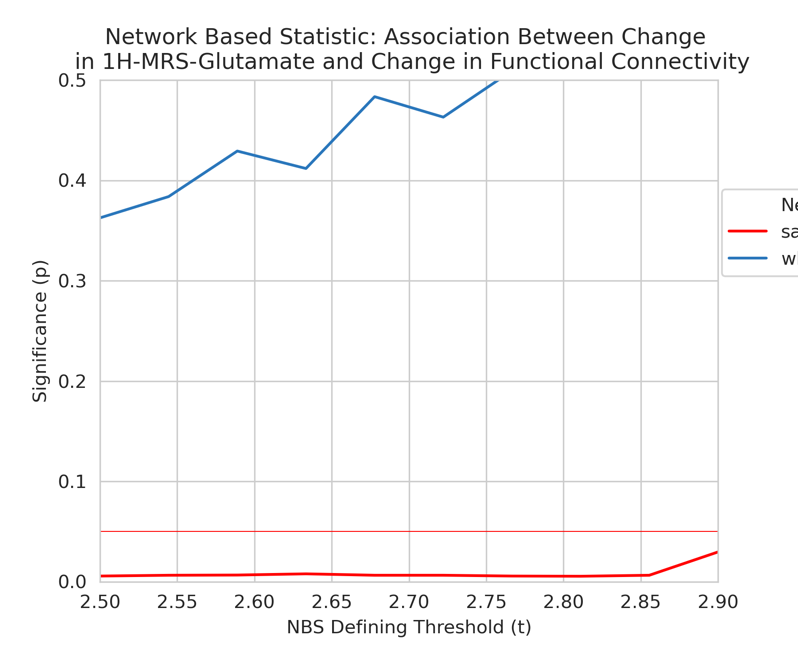


Whole Brain

Salience Network

### eFigure 5. Study B association between change in glutamate and change in connectivity after controlling for age and sex

Change in functional connectivity is positively associated with frontal cortex glutamate concentrations when nodes are restricted to the salience network

The NBS threshold of t=2.5-2.9 is relatively stringent – preserving only connections that show an association with glutamate at a level of statistical significance greater than p<0.01-0.005. When relaxing the threshold to t=2.0 (equivalent top<0.05) the results remain similar:


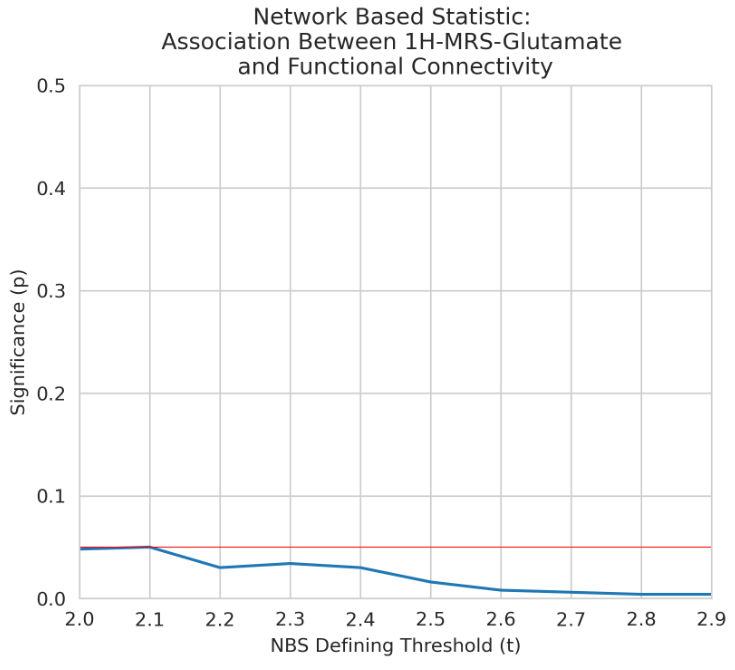


### eFigure 6. Study A: Glutamate associated networks at wider range of NBS thresholds

Higher anterior cingulate glutamate concentrations are associated with reduced connectivity across a range of NBS thresholds. The horizontal red line represents the p<0.05 threshold while the blue line represents statistical significance across a range of thresholds.


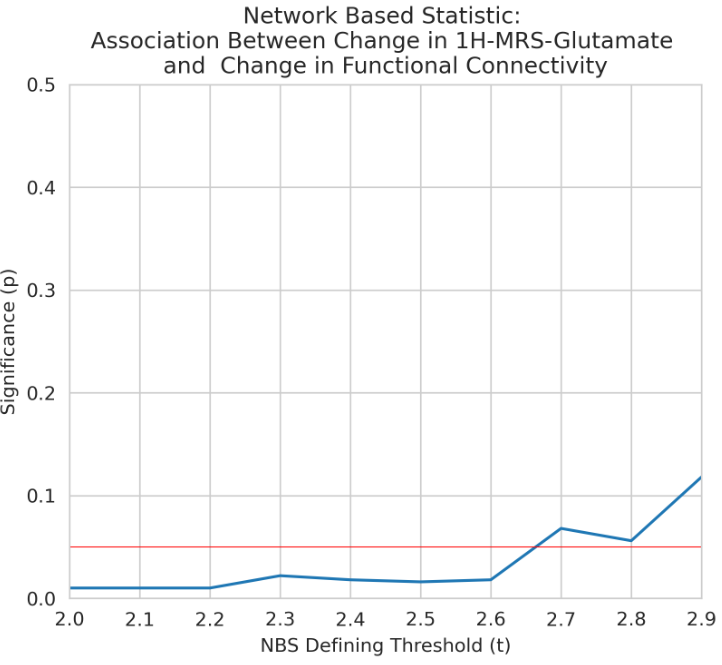


### eFigure 7. Study B: Glutamate associated networks at wider range of NBS thresholds

When analysis is constrained to the salience network, at certain NBS thresholds there is a positive association between change in glutamate concentrations and change in connectivity following riluzole administration. The horizontal red line represents the p<0.05 threshold, the blue line represents statistical significance when analysis is constrained to the salience network.

# References

1 Beck K, Mccutcheon R, Bloomfield MAP, Gaughran F, Reis Marques T, Maccabe J *et al.* The practical management of refractory schizophrenia - the Maudsley Treatment REview and Assessment Team service approach. *Acta Psychiatr Scand* 2014; **130**. doi:10.1111/acps.12327.

2 Jones SH, Thornicroft G, Coffey M, Dunn G. A brief mental health outcome scale-reliability and validity of the Global Assessment of Functioning (GAF). *Br J Psychiatry* 1995; **166**: 654–659.

3 McCutcheon R, Beck K, D’Ambrosio E, Donocik J, Gobjila C, Jauhar S *et al.* Antipsychotic plasma levels in the assessment of poor treatment response in schizophrenia. *Acta Psychiatr Scand* 2018; **137**: 39–46.

4 Howes OD, McCutcheon R, Agid O, de Bartolomeis A, van Beveren NJ, Birnbaum ML *et al.* Treatment-Resistant Schizophrenia: Treatment Response and Resistance in Psychosis (TRRIP) Working Group Consensus Guidelines on Diagnosis and Terminology. *Am J Psychiatry* 2017; **174**: 216–229.

5 Gasparovic C, Bedrick EJ, Mayer AR, Yeo RA, Chen H, Damaraju E *et al.* Test-retest reliability and reproducibility of short-echo-time spectroscopic imaging of human brain at 3T. *Magn Reson Med* 2011; **66**: 324–332.

6 Gasparovic C, Song T, Devier D, Bockholt HJ, Caprihan A, Mullins PG *et al.* Use of tissue water as a concentration reference for proton spectroscopic imaging. *Magn Reson Med* 2006; **55**: 1219–1226.

7 LCModel’s home page. http://s-provencher.com/ lcmodel.shtml.

8 Esteban O, Markiewicz CJ, Blair RW, Moodie CA, Isik AI, Erramuzpe A *et al.* fMRIPrep: a robust preprocessing pipeline for functional MRI. *Nat Methods* 2019; **16**: 111–116.

9 Gorgolewski K, Burns CD, Madison C, Clark D, Halchenko YO, Waskom ML *et al.* Nipype: A Flexible, Lightweight and Extensible Neuroimaging Data Processing Framework in Python. *Front Neuroinform* 2011; **5**. doi:10.3389/fninf.2011.00013.

10 Avants BB, Tustison N, Song G. Advanced Normalization Tools (ANTS). *Insight J* 2009; : 1–35.

11 Tustison N, others. N4ITK: Improved N3 Bias Correction. *Ieee Tmi* 2010; **29**: 1310–1320.

12 Zhang Y, Brady M, Smith S. Segmentation of brain MR images through a hidden Markov random field model and the expectation-maximization algorithm. *IEEE Trans Med Imaging* 2001; **20**: 45–57.

13 Dale AM, Fischl B, Sereno MI. Cortical surface-based analysis: I. Segmentation and surface reconstruction. *Neuroimage* 1999; **9**: 179–194.

14 Klein A, Ghosh SS, Bao FS, Giard J, Häme Y, Stavsky E *et al.* *Mindboggling morphometry of human brains*. 2017 doi:10.1371/journal.pcbi.1005350.

15 Wang S, Peterson DJ, Gatenby JC, Li W, Grabowski TJ, Madhyastha TM. Evaluation of field map and nonlinear registration methods for correction of susceptibility artifacts in diffusion MRI. *Front Neuroinform* 2017; **11**: 1–9.

16 Treiber JM, White NS, Steed TC, Bartsch H, Holland D, Farid N *et al.* Characterization and correction of geometric distortions in 814 Diffusion Weighted Images. *PLoS One* 2016; **11**: 1–9.

17 Greve DN, Fischl B. Accurate and robust brain image alignment using boundary-based registration. *Neuroimage* 2009; **48**: 63–72.

18 Jenkinson M, Bannister P, Brady M, Smith S. Improved optimization for the robust and accurate linear registration and motion correction of brain images. *Neuroimage* 2002; **17**: 825–841.

19 Cox RW, Hyde JS. Software Tools for Analysis and Visualization of FMRI Data NMR in Biomedicine, in press. *NMR Biomed* 1997; **10**: 171–8.

20 Lanczos C. Evaluation of Noisy Data. *J Soc Ind Appl Math Ser B Numer Anal* 1964; **1**: 76–85.

21 Abraham A, Pedregosa F, Eickenberg M, Gervais P, Muller A, Kossaifi J *et al.* Machine Learning for Neuroimaging with Scikit-Learn. 2014; **8**: 1–10.

22 Ciric R, Rosen AFG, Erus G, Cieslak M, Adebimpe A, Cook PA *et al.* Mitigating head motion artifact in functional connectivity MRI. *Nat Protoc* 2018; **13**: 2801–2826.

23 Pillinger T, Rogdaki M, McCutcheon RA, Hathway P, Egerton A, Howes OD. Altered glutamatergic response and functional connectivity in treatment resistant schizophrenia: the effect of riluzole and therapeutic implications. *Psychopharmacology (Berl)* 2019; **236**: 1985–1997.

24 Kundu P, Brenowitz ND, Voon V, Worbe Y, Vértes PE, Inati SJ *et al.* Integrated strategy for improving functional connectivity mapping using multiecho fMRI. *Proc Natl Acad Sci U S A* 2013; **110**: 16187–16192.

25 Whitfield-Gabrieli S, Nieto-Castanon A. Conn: A Functional Connectivity Toolbox for Correlated and Anticorrelated Brain Networks. *Brain Connect* 2012; **2**: 125–141.

26 Freedman D, Lane D. A nonstochastic interpretation of reported significance levels. *J Bus Econ Stat* 1983; **1**: 292–298.

27 Zalesky A, Cocchi L, Fornito A, Murray MM, Bullmore E. NeuroImage Connectivity differences in brain networks. *Neuroimage* 2012; **60**: 1055–1062.

28 Gordon EM, Laumann TO, Adeyemo B, Huckins JF, Kelley WM, Petersen SE. Generation and Evaluation of a Cortical Area Parcellation from Resting-State Correlations. *Cereb Cortex* 2016; **26**: 288–303.
